# Gait, falls, cognitive function, and health-related quality of life after shunt-treated idiopathic normal pressure hydrocephalus—a single-center study

**DOI:** 10.1007/s00701-022-05309-4

**Published:** 2022-07-13

**Authors:** Caroline Hallqvist, Helena Grönstedt, Lisa Arvidsson

**Affiliations:** 1 Women’s Health and Allied Health Professionals Theme, Medical Unit Occupational Therapy and Physiotherapy, Karolinska University Hospital, Stockholm, Sweden; 2 Department of Clinical Neuroscience, Karolinska Institutet, Solna, Stockholm Sweden; 3 Department of Neurosurgery, Karolinska University Hospital, Eugeniavägen 27, Karolina Tower Hotel plan 4, 171 76 Stockholm, Sweden

**Keywords:** Gait ability, Falls, Cognition, Health-related quality of life, Idiopathic normal pressure hydrocephalus, Shunt surgery

## Abstract

**Background:**

Normal pressure hydrocephalus (NPH) is a neurological disorder, characterized by gait- and balance disturbance, cognitive deterioration, and urinary incontinence, combined with ventricular enlargement. Gait ability, falls, cognitive status, and health-related quality of life pre and post surgery have not previously been studied at Karolinska University Hospital.

**Methods:**

One hundred and eighteen patients with iNPH that underwent shunt surgery at Karolinska University Hospital during the years from 2016 to 2018 were included. Results of walking tests, test for cognitive function, and self-estimated health-related quality of life, before and 3 months after surgery, were collected retrospectively as a single-center study.

**Results:**

Walking ability, cognitive function, and health-related quality of life significantly increased 3 months after shunt surgery. A positive significant correlation was seen between a higher self-estimated quality of life and walking ability.

**Conclusions:**

Patients with suspected iNPH treated with shunt surgery at Karolinska University Hospital improved their walking ability and cognitive functioning 3 months after shunt surgery. A positive significant correlation was seen between a higher self-estimated quality of life and walking ability but not with increased cognitive function. We then concluded that the selection of patients for shunting maintained a high standard.

## Introduction

Normal pressure hydrocephalus (NPH) is a neurological disorder, characterized by gait- and balance disturbance, cognitive deterioration, and urinary incontinence, combined with ventricular enlargement and a moderately elevated lumbar cerebrospinal fluid (CSF) [1]. While the cause of secondary NPH is known, the etiology of idiopathic NPH (iNPH) is unknown although vascular comorbidity has been proposed as a possible cause with risk factors as high blood pressure and diabetes [6]. The majority of patients are 70–80 years of age [27]. Patients suffering from iNPH are most commonly treated by diversion of CSF through a ventriculoperitoneal shunt [26]. If untreated, the hazard ratio for early death is 3.8 [16].

iNPH is characterized by a classical triad of symptoms: gait and balance disturbance, urinary incontinence, and cognitive decline. The cardinal symptom is the gait disturbance. The gait is typically broad-based and shuffling due to decreased velocity and step length as well as step height. Retropulsion and freezing of gait phenomena are also common [2]. In the early stages, gait changes can be insignificant, with progression over time. These disturbances often lead to an increased risk of falling combined with fear of falling that may contribute to loss of independence [10]. The cognitive deficits seen in iNPH are characterized by deficits in memory, visuospatial abilities, psychomotor speed, and executive function. For patients with cognitive deficits, the inability to perform activities of daily life (ADL) is the main reason of deteriorated health-related quality of life (HRQoL). Before shunting, many patients with iNPH, struggle with their ADL, have depressive symptoms, and estimate their HRQoL lower than the general population [17].

## Aim

Gait ability, falls, cognitive status, and health-related quality of life pre and post surgery have not previously been studied at Karolinska University Hospital. Our aim was to investigate how these parameters varied, as well as correlated, in INPH patients evaluated by CSF tap test, selected for shunt surgery, and evaluated 3 months post surgery at Karolinska University Hospital.

## Methods and materials

Data regarding Karolinska University Hospital were extracted from The Swedish Neuro Registries—a national quality registry which aims to improve the equity and quality of neurological care as well as ensure adherence to national guidelines. If data were missing in the registry, they were extracted from medical records.

The Timed Up and Go test (TUG) [22] and 10-m walk test (10MWT) [4] were used as a measure of gait. TUG is a reliable and valid test for quantifying functional gait and may also be useful in following a clinical change over time [22]. The 10MWT has a great test–retest reliability and good validity and is the most preferable short walk test in clinical research. It is also a good indicator of measuring health status [11]. Gait velocity in 10MWT was recalculated to meters per second (m/s) and used for analyses.

Cognitive status was assessed by the Mini-Mental State Examination (MMSE, range 0–30) [8]. The test is considered to have a satisfactory reliability and validity [25].

The 5-level EQ-5D version (EQ-5D-5L) was used for assessment of self-rated health-related quality of life. EQ-5D-5L is a reliable and valid generic instrument that describes health status in five domains. The EQ-5D visual analog scale (EQ VAS) was used to provide a global assessment of the patient’s health from 0 (worst imaginable health) to 100 (the best imaginable health) [7].

Falls within the last 12 months, pre surgery, and 3 months post surgery were documented in the patient’s medical record. One fall or more were registered by “Yes” in the data set and no falls reported as “No.”

All tests were performed before (pre-CSF tap test) and 3 months after ventriculo-peritoneal shunt surgery. If the elapse time for surgery was more than 3 months, the patients were revaluated preoperatively.

### Participants

**Fig. 1:**
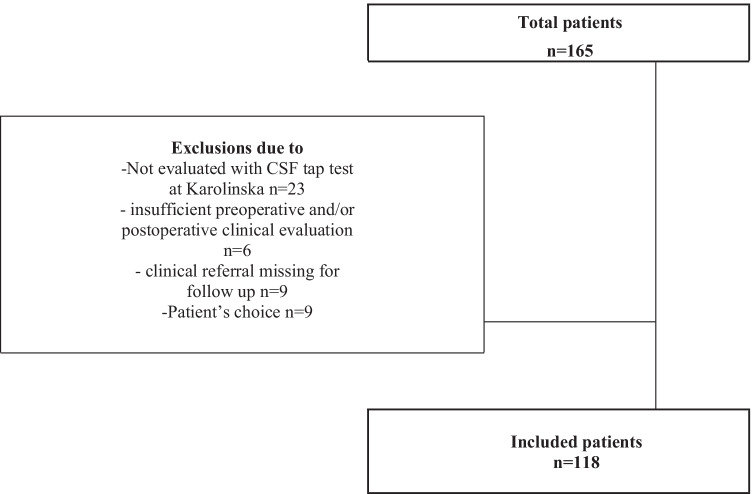
Study flowchart

Patients diagnosed with iNPH, tap tested and underwent shunt surgery during the years of 2016–2018 at Karolinska University Hospital, were included. Patients that lacked results of gait pre- and postoperative, were excluded but no patients were excluded due to cognitive status. The flowchart of the study is shown in Fig. 1.

### Statistical analysis

All statistical analyses were conducted with IBM SPSS Statistics, version 25. Statistical significance was set at the 0.05-level. Due to the lack of normally distributed variables, we used mainly non-parametric statistics.

The Wilcoxon signed-rank test was used in TUG, 10MWT, MMSE, and EQ-5D-5L to determine whether there was a significant difference between the median values in the two related groups.

The dependent *T* test was used in EQ VAS to determine whether there was a statistically significant difference between the means in the two related groups.

Spearman’s rank-order correlation was used to analyze the degree of association between the variables 10MWT, TUG, MMSE, and EQ VAS after surgery. To calculate EQ-5D-5L index, the EQ-5D-5L crosswalk index value calculator with the United Kingdom algorithm was used.

### Ethical approval

The study was approved by the Regional ethical review board in Stockholm, DNR 2019–00,353.

## Results

### Demographics

One-hundred and sixty-five patients underwent shunt surgery at Karolinska University Hospital during the years 2016–2018. Forty-seven of the patients were excluded due to exclusion criterias, and the remaining 118 patients were included in the study (Fig. 1).

Mean age was 73.5 years and 56% (*n* = 66) were men. Mean time from neurological examination to surgery was 5.2 months (± 3.4–7.2), and time from decision of surgery to shunt surgery was 2.6 months (± 1.4–3.6). The majority got the same model of an adjustable shunt (*n* = 93). Hypertension (64%), cardiovascular disease (29%), and diabetes (23%) were the most common comorbidities. Spinal stenosis, that can affect gait and balance, occurred in only 6% of the patients.

### Gait

The 10MWT was initially evaluated at Karolinska University Hospital in spring 2016; therefore, 22 of the 118 patients were excluded. Additionally, five patients were not able to complete the test pre and/or post surgery and for one patient data were missing. In total, 90 patients remained for analysis. A statistically significant increase (*p* < 0.0001) was seen in median walking speed that improved with 25%, from 0.72 m/s (0.54–0.935) to 0.9 m/s (0.68.9–1.05), and the number of steps decreased significantly (*p* < 0.0001) from 23 steps (19–30) to 20 steps (17–24), see Table 2. Forty-eight patients (53.3%) improved their walking speed with more than 0.1 m/s, and 71 patients (78.9%) walked with fewer steps postoperatively.

### Functional gait

Six patients did not complete the test neither pre and/or post surgery. In total, 112 patients remained for data analysis.

TUG median time significantly decreased (*p* < 0.0001) from 19 (14.27–24.87) to 15.25 s (12.35–19.5) and for 72 (62.5%) patients, the improvement was > 2.5 s. The TUG median number of steps decreased significantly from 23 (33–19) to 21 (25–19) (Table 1).

**Table 1:** The change of 10MWT, TUG, and MMSE pre and 3 months post surgery

Test		Median (min–max)		*p* value
	*n*	Pre	Post	
10MWT m/s	90	0.72 (0.1–1.33)	0.9 (0.25–1.49)	< 0.0001
10MWT steps	89	23 (12–99)	20 (14–63)	< 0.0001
TUG sec	112	19 (7.2–187)	15.25 (7.6–128)	< 0.0001
TUG steps	112	23 (12–210)	21 (13–100)	< 0.0001
MMSE	100	24 (0–30)	26 (7–30)	< 0.0001

*10MWT* 10-m walk test, *TUG* timed up and go, *MMSE* Mini-Mental State Examination.

### Cognitive status

Due to limited resources, 18 patients did not accomplish MMSE pre surgery, and 100 patients remained for data analysis.

Median score on MMSE increased statistically significant (*p* < 0.0001) from median 24 to 26 points (Table 1). A result of ≥ 24 points are to be considered as normal cognition [25].

### Health-related quality of life

Due to the fact that the EQ-5D-5L version was included in the test battery at Karolinska University Hospital from February 2017, 42 patients were excluded. Data were missing for another 12 and in total, 64 patients remained for analysis.

EQ-Index increased (*p* = 0.00004) from median 0.684 (0.09 min–0.863 max) to 0.732 (0.343 min–1.0 max) and EQ VAS mean from 59.738 (21.428) to 68.150 (19.966) (*p* = 0.0026). The patients reported improved HRQoL in all individual parameters: mobility, self-care, usual activity, pain/discomfort and anxiety/depression. The greatest improvement was seen in the category mobility, and the smallest increase was seen in the category of pain/discomfort (Table 2).

**Table 2:** Distribution of EQ-5D-5L dimension responses pre and 3 months post surgery

Dimension	Preoperative	Postoperative	*p* value
	***n* (%)**	***n* (%)**	
**Mobility**			*m* < 0.0001
No problems	6 (9.5%)	20 (31%)	
Slight problems	15 (23.5%)	17 (26.5%)	
Moderate problems	25 (39%)	26 (40.5%)	
Severe problems	18 (28%)	1 (1.5%)	
Unable to walk about	0 (0%)	0 (0%)	
**Self-care**			0.0019
No problems	33 (52%)	47 (73.5%)	
Slight problems	17 (26.5%)	10 (15.5%)	
Moderate problems	9 (14%)	6 (9.5%)	
Severe problems	4 (6%)	0 (0%)	
Unable to wash or dress	1 (1.5%)	1 (1.5%)	
**Usual activities**			0.0035
No problems	15 (23%)	19 (29.5%)	
Slight problems	16 (25%)	23 (36%)	
Moderate problems	12 (19%)	14 (22%)	
Severe problems	13 (20%)	6 (9.5%)	
Unable to do usual activities	8 (13%)	2 (3%)	
**Pain/discomfort**			0.402
No pain/discomfort	19 (30%)	22 (34.5%)	
Slight pain/discomfort	19 (30%)	15 (23.5%)	
Moderate pain/discomfort	22 (34%)	23 (36%)	
Severe pain/discomfort	3 (4.5%)	4 (6%)	
Extreme pain/discomfort	1 (1.5%)	0 (0%)	
**Anxiety/depression**			0.0037
Not anxious/depressed	22 (34.5%)	27 (42%)	
Slightly anxious/depressed	22 (34.5%)	25 (39%)	
Moderately anxious/depressed	14 (22%)	10 (15.5%)	
Severely anxious/depressed	4 (6%)	2 (3%)	
Extremely anxious/depressed	2 (3%)	0 (0%)	

## Correlations

There was a significant correlation (*p* < 0.05) between post tap test and post shunt results of TUG (sec) (Spearman’s rho 0.764) and 10MWT (m/s) (Spearman’s rho 0.818). The results also showed that increased walking speed measured by TUG (sec) and 10MWT (m/s) correlated with a higher score on EQ VAS (*p* < 0.05) (Spearman’s rho − 0.255 and 0.251) but no correlation between MMSE and EQ VAS was seen.

### Falls after surgery

Eighty patients (68%) had a history of falls 12 months pre surgery; there was no significant reduction of falls post surgery. Two patients had fallen post surgery but not pre surgery. Thirty-six patients (30.5%) had no falls at all.

In the non-faller group, there was a significant correlation (*p* < 0.05) in time and number of steps on both 10MWT and TUG (Spearman’s rho − 0.286, − 238, − 0.224, − 224), as well as a higher score on MMSE post surgery (0.210). No significant correlation was seen with EQ VAS. There was no correlation between 10MWT, TUG, MMSE, and EQ VAS in the faller group.

## Discussion

In this single-center study, we demonstrated a significant improvement in gait ability, cognitive status, and HRQoL 3 months after shunt surgery due to iNPH. Our results indicated a good selection of patients for shunt treatment at Karolinska University Hospital.

Gait ability was investigated using two instruments with good reliability, validity, and responsiveness for patients with iNPH. The instruments are easy to use in a clinical practice, and the 10MWT is considered the best instrument to assess gait ability [11].

One weakness with 10MWT and TUG is their ceiling effect, i.e., for a patient with mild gait disturbance, it may be difficult to measure a clinical difference over time. The patients had, despite the shunt surgery, a slower walking speed on 10MWT and a poorer performance on TUG in comparison with the reference values for healthy elders [4, 5].

Previous studies suggests that a TUG time reduced by 2.5 s after an intervention can be considered a clinical improvement [3]. Our results showed a median difference of 3.75 s post surgery. This is in line with the results presented in other studies [2, 23].

Only a few studies have been focusing on falls of iNPH patients [19, 21]. Falls affect not only the individual but also healthcare and the society. Nikaido et al. [21], could demonstrate that postural instability improved as soon as 1 week after surgery and Larsson et al. [19], demonstrated that the decreased number of falls sustained for as long as 12 months post surgery. In our study, the postoperative observational time was only 3 months but at 12 months preoperative, and no statistical comparison was performed due to the differences in observational time.

Gait velocity and step-length have been shown to be reduced among fallers in comparison to non-fallers in patients with iNPH [21]. These results were also seen in our study. We also found a correlation between an increased functional status in the non-faller group post surgery. This suggests that an improvement in both gait- and cognitive status may lead to a decreased risk of falling. In contradiction, Takeuchi and Yajima showed an increased risk of falls in the first 6 months post shunt surgery, even if there was an improvement in both gait and cognitive function [24].

Cognitive status improved assessed by the MMSE post surgery. MMSE is frequently used to assess cognitive function in iNPH [18, 20]. We did not exclude patients due to dementia as no patients was excluded from shunt surgery due to low cognitive function. The study sample would then be more representative for patients with iNPH.

The patients’ median value on the EQ-index improved from 0.684 pre surgery to 0.732 post surgery. The patients improved in all five parameters: mobility, self-care, usual activity, pain/discomfort, and anxiety/depression. The greatest improvement was seen in the category mobility, and the least alteration was in the category pain/discomfort. This is in line with the findings by Hülser et al. [13], despite the fact that our study group walked faster in 10MWT pre surgery, but the percentage of the improvement was equal in both studies. Our study aimed on a 3-month follow-up. Larsson et al. demonstrated an increased HRQoL 12 months after shunt surgery. With a longer follow-up time period. we might have captured further improvement, but this was not the focus of this study.

The INPH patients had an overall lower HRQoL in all subcategories than the general elderly population [9, 12]. This may be due to the fact that many iNPH patients also suffer from long-term comorbidities and limited social interaction due to the classical iNPH symptoms.

We found a correlation between increased functional gait and gait ability and a higher estimated quality of life (EQ VAS). Increased mobility may lead to less dependence to other people which in turn is connected to decreased risk of depression and therefore a higher quality of life [15].

Surprisingly, no correlation was found between MMSE and EQ VAS. Even through the patient increased their cognitive status post surgery, they did not estimate an increased quality of life. Since no patients with dementia were excluded as in other studies [14, 15], it is a possibility that patients post surgery are more aware of their situation, and therefore grade themselves lower in EQ-5D-5L.

These results may be due to disability to understand the questions pre surgery or not the accurate measuring instrument for iNPH patients.

Future studies would focus on pre- and postoperative effects of physical training that is specifically developed for patients with iNPH.

This is a single-center study; no examiner was blinded, giving room for bias. However, the test process was standardized, the surgery was performed by a small group of trained neurosurgeons, and the tests were performed by qualified clinicians specialized in evaluating patients with iNPH.

The clinical awareness of iNPH has risen, and the number of shunt surgeries has increased, and our experience is that patients are diagnosed and treated earlier than before. We conclude that the clinical evaluation used at Karolinska University Hospital well discriminates for positive results of shunt surgery.

## Conclusion

This is the first study to evaluate shunt surgery for patients with iNPH at Karolinska University Hospital. The results showed, in line with previous studies, an improvement in gait ability. A positive significant correlation was seen between a higher self-estimated quality of life and walking ability but not between self-estimated quality of life and increased cognitive function.

## Data Availability

The original data can be requested from the Swedish Hydrocephalus Quality Registry and patient journals.

## Abbreviations

ADL: Activities of daily life
CSF: Cerebrospinal fluid
EQ-5D-5L: The 5-level EQ-5D version
EQ-Index: EQ-5D-5L crosswalk index value
EQ VAS: The EQ-5D visual analog scale
HRQoL: Health-related quality of life
iNPH: Idiopathic normal pressure hydrocephalus
MMSE: Mini-Mental State Examination
NPH: Normal pressure hydrocephalus
TUG: Timed Up and Go test
10MWT: 10-Meter walk test
